# A scoping review of theoretical and measurement approaches to women’s empowerment in low-and middle-income countries’ capacity-building interventions

**DOI:** 10.1080/16549716.2026.2676412

**Published:** 2026-05-26

**Authors:** Fitsum Kibreab, Asia Haraburda, Yousef A. A. Albaba, Ai Chee Yong, Kajal Shah, Sanele Buthelezi, Ving Fai Chan

**Affiliations:** a Health Research and Resources Centre Division, Ministry of Health, Asmara, Eritrea; b Centre for Public Health, Queens University Belfast, Belfast, UK; c School of Physics, Clinical and Ophthalmic Sciences, Dublin Institute of Technology, Dublin, Ireland; d Department of Optometry, Faculty of Health Sciences, University of Johannesburg, Johannesburg, South Africa

**Keywords:** Entrepreneur, microfinance, sustainable development, women’s health, capacity building

## Abstract

Women’s empowerment is critical for achieving gender equality and sustainable development in low-and-middle-income countries (LMICs). Capacity-building interventions, such as vocational training, microfinance, and digital literacy programmes, are frequently employed to foster empowerment. However, wide variation exists in how empowerment is defined, theorised, and measured, limiting comparability across studies. This scoping review mapped the conceptual foundations, theoretical frameworks, and measurement approaches used in capacity-building interventions for women entrepreneurs in LMICs. The review followed Arksey and O’Malley’s framework and was reported in line with PRISMA-ScR guidelines. Searches were conducted in PubMed, Scopus, Web of Science, and JSTOR, complemented by grey literature from major development agencies (2010–2024). Eligible studies focused on capacity-building interventions targeting women entrepreneurs in LMICs. Two reviewers independently screened and extracted data, which were synthesised thematically. Of 11,109 records identified, 25 met inclusion criteria. Most studies were cross-sectional, conducted in sub-Saharan Africa (48%) and South Asia (32%). While 76% defined empowerment, 60% did not employ any explicit theoretical or conceptual framework. Among those that did, frameworks, such as Kabeer’s, Longwe’s, and Malhotra et al.’s were used inconsistently. Economic empowerment was the most frequently assessed domain, often measured through income or business performance, while social, political, and psychological dimensions were seldom examined. Indicators were heterogeneous and largely self-reported. Conceptual ambiguity and inconsistent measurement hinder progress in women’s empowerment research in LMICs. Future studies should adopt theoretically grounded, multidimensional, and context-sensitive frameworks to capture empowerment as a dynamic process beyond economic gains.

## Paper Context

- **Main findings:** Capacity-building interventions for women’s empowerment in low-and-middle income countries use highly heterogeneous and often atheoretical definitions and indicators, with a dominant focus on economic outcomes and limited attention to social, political, psychological, or process-based dimensions of empowerment.
- **Added knowledge:** This review systematically maps how women’s empowerment is conceptualised, theorised, and measured across capacity-building interventions in low-and-middle income countries, demonstrating a persistent disconnect between empowerment theory and evaluation practice and highlighting gaps in multidimensional and longitudinal measurement.
- **Global health impact for policy and action:** Policymakers, funders, and implementers should move beyond income-only metrics and adopt theoretically grounded, multidimensional, and context-sensitive empowerment frameworks to design, monitor, and scale equitable capacity-building programmes aligned with Sustainability Development Goal 5.

## Background

### The importance of women’s empowerment

Women’s empowerment – the process of enhancing an individual’s or group’s capacity to make purposive choices and to transform those choices into desired actions and outcomes [1] – is widely recognised as essential in achieving gender equality and sustainable development in low-and-middle-income countries (LMICs) [2]. In these settings, barriers to women’s decision-making and agency are deeply rooted in patriarchal traditions, limited access to formal education and employment, and legal or policy constraints [3]. Women’s empowerment should therefore be viewed not just as a symbolic outcome, but a mechanism for change and tangible improvements in well-being. Empowered women are more likely to participate in the labour force [4], lead their communities [5], access education [6] and experience better health outcomes [7]. These benefits extend beyond the individual as evidence shows women reinvest their earnings in family well-being, children’s education, and community development [8].

### Capacity-building as a tool for women’s empowerment

Capacity-building is one of the most common strategies employed to foster women’s empowerment in LMICs [9]. The United Nations (UN) define capacity-building as ‘the process of developing and strengthening the skills, instincts, abilities, processes and resources that organizations and communities need to survive, adapt, and thrive in a fast-changing world’ [10]. In practice, capacity-building interventions for women span a wide range of activities including vocational and digital skills training [11], health education [12], and entrepreneurship [13]. Capacity–building interventions provide a particularly powerful avenue for women’s empowerment in LMICs because they intersect economic, social, and psychological domains. For many women, entrepreneurship and access to personal finance represent one of the few viable routes to autonomy and social recognition in contexts where formal employment opportunities remain limited [14]. Yet, women face many systemic barriers, such as unequal access to loans, markets, digital tools, and networks, limiting their economic participation [15]. Capacity-building programmes often report improvements in economic or social indicators; however, it must be noted that their effects are mediated through empowerment processes – including enhanced agency, confidence, resource access, and participation in economic and civic life [16–18]. Understanding how empowerment is conceptualised and measured within capacity-building interventions is therefore essential for identifying what works, for whom, and under what circumstances.

In LMIC contexts, examining empowerment as a pathway rather than merely an outcome is particularly important. Capacity-building initiatives frequently operate within environments characterised by entrenched gender norms, informal labour markets, limited social protection, and restricted access to assets, credit, and digital infrastructure. In such settings, improvements in income, training, or business performance do not automatically translate into sustained wellbeing, autonomy, or equity. The extent to which these gains are converted into lasting social and economic advancement depends on whether women are able to exercise agency, influence household and community decision-making, control resources, and navigate structural constraints. Understanding empowerment as the mechanism through which capacity-building translates into broader development outcomes is therefore critical. Without explicit attention to this pathway, interventions risk measuring short-term economic outputs, while overlooking the deeper relational and structural transformations necessary for durable gender equity in LMICs.

### Conceptual ambiguity of women’s empowerment

Despite the proliferation of capacity-building programmes, significant variability exists in how empowerment is defined and operationalised across studies. Many interventions present empowerment as an intended outcome but fail to clearly articulate the theoretical or conceptual underpinnings that guide their approach. The term is often used loosely or inconsistently, sometimes framed as an individual psychological trait [19], other times as a relational [20] or structural process [21], without adequate reflection on its multidimensional or context-specific nature. Some studies focus narrowly on economic indicators, such as income or employment [22,23], while others examine self-efficacy, voice, or decision-making [17,20,24]. These divergent approaches limit our ability to assess effectiveness, draw comparisons, or synthesise lessons across contexts.

A further complication arises from the frequent conflation of ‘women’s empowerment’ with related but distinct constructs, such as gender equality, gender equity, and women’s rights [16,20,22]. While these concepts are interconnected, failing to distinguish between them can obscure the aims of an intervention and undermine the design of appropriate evaluation frameworks. For instance, promoting equal access to resources (gender equality) does not necessarily ensure that women gain the power to make meaningful choices (empowerment). Clear distinctions between these concepts are essential to define intervention goals, select appropriate indicators, and interpret outcomes accurately.

Empowerment is inherently complex and influenced by intersecting factors, such as sociocultural norms, institutional structures, education levels, age, ethnicity, and geographical location. Consequently, the relevance and impact of a given intervention may vary significantly by setting. Recognising this, scholars have called for greater attention to context-specific frameworks that reflect the lived realities of women in different regions, cultures, and life stages [25].

### Theoretical frameworks

To address these issues, several theoretical and measurement frameworks have been developed over the past two decades. One of the most influential is Kabeer’s framework which conceptualises empowerment as a process involving three interrelated dimensions: resources (precondition), agency (process), and achievements (outcomes) [20]. This framework offers a useful lens through which to design and evaluate interventions by linking material, cognitive, and relational factors. Other frameworks, such as the Women’s Empowerment in Agriculture Index (WEAI), provide validated tools to measure empowerment in specific contexts [26]. In the policy space, the British Equality and Human Rights Commission’s Measurement Framework for Equality and Human Rights [27] is used to assess equality of opportunity and outcome. However, such frameworks, especially those developed in high-income countries, may face challenges in applicability, complexity, and cultural relevance when used in LMICs [28]. Despite the availability of these frameworks, there is limited understanding of how often, and in what ways, they are applied in capacity-building interventions for women’s empowerment in LMICs.

### Measurement challenges

Efforts have been made to develop tools such as the EMERGE framework (evidence-based measures of empowerment for research on gender equality) [29], which acts as a repository of rigorous, context-sensitive, and validated indicators for gender equality and empowerment research, but measurement remains a particularly challenging aspect of empowerment research. Studies often rely on subjective self-reported indicators (e.g. confidence or satisfaction), indirect proxies (e.g. asset ownership), or oversimplified metrics (e.g. income generation) that fail to capture the full breadth of empowerment processes [29]. Moreover, many evaluations prioritise quantitative indicators without incorporating qualitative methods that explore women’s lived experiences, perceptions of agency, or social relationships [20]. These limitations hinder the ability to assess if empowerment has occurred, its scope and for whom it was achieved.

### Knowledge gap and review aims

Existing reviews tend to focus on sectoral outcomes (e.g. health, education, agriculture) or programme types (e.g. microfinance, vocational training) but rarely interrogate the conceptual foundations or theoretical coherence of the interventions themselves [30]. Previous work has also examined measurement approaches for community capacity-building [31]. Capacity-building was assessed through learning opportunities, skills development, and resource mobilisation, while economic empowerment was captured through mobility, access to savings, and participation in decision-making. However, no previous work has described theoretical and conceptual foundations and measurement approaches to women’s empowerment in capacity-building interventions in LMICs. This scoping review aims to fill the gap by

- assessing the conceptual foundations of published capacity-building interventions for women’s empowerment among women entrepreneurs in LMICs,
- identifying whether and how theoretical frameworks are employed to guide intervention design and evaluation, and
- mapping and categorising the indicators used to measure empowerment and its impact.

Through this, the review aims to promote greater conceptual clarity, highlight promising practices, and support more coherent, comparable, and contextually grounded approaches to designing and evaluating empowerment interventions in future research and intervention design.

## Methods

Prior to conducting this review, a study protocol was developed to guide the search, screening, and synthesis processes in accordance with established scoping review methodology (Arksey & O’Malley [32]; Tricco et al. [33]). Given the breadth and complexity of the literature identified, the review was structured as a three-paper series. This decision reflects both the volume and breadth of data and the need to provide sufficient analytical depth to each dimension of the evidence base. This approach is consistent with academic conventions in systematic and scoping review publication, where a single synthesis would otherwise become unwieldy and risk superficial coverage of key themes. Each paper engages critically with distinct but complementary aspects of the evidence base, and together, the series provides a comprehensive map of capacity-building initiatives for women entrepreneurs in LMICs, their effectiveness, and the conceptual and methodological frameworks underpinning them.

- Paper 1 [34] develops a taxonomy of intervention types, categorising models, such as microfinance, digital platforms, community-based cooperatives, and vocational training. It examines how these initiatives are designed and delivered and analyses the barriers and enablers that shape their uptake, accessibility, and sustainability. Thirty studies, predominantly from sub-Saharan Africa and South Asia, show that the most common intervention types are microfinance and financial services, vocational training, multi-sectoral ‘mixed’ models, digital initiatives, entrepreneurship programmes, political participation initiatives and cash-transfer schemes. Interventions integrating financial access with skills development and explicit empowerment components demonstrated more robust and sustainable outcomes than single-focus approaches. Common barriers included entrenched gender norms, resource constraints, and digital exclusion. Overall, capacity-building programmes strengthened women’s social and economic agency and contributed indirectly to improved household and community wellbeing, supporting multiple Sustainable Development Goals.
- Paper 2 (in preparation) shifts the focus to outcomes, asking what works, where, and how well. It synthesises evidence on effectiveness, geographical variation, and the methodological quality of existing evaluations.
- Paper 3, which is the focus of the present article, examines the conceptual foundations of interventions. It analyses the theoretical frameworks employed and maps the indicators used to measure empowerment and impact, with the goal of advancing conceptual clarity and promoting consistency in the measurement of women’s empowerment.

The scoping review was conducted in line with the Joanna Briggs Institute (JBI) methodology [35] and reported according to the Preferred Reporting Items for Systematic Reviews and Meta-Analyses extension for Scoping Reviews (PRISMA-ScR) guidelines [33], ensuring systematic and transparent reporting. In addition, the Arksey and O’Malley framework [32] was used to structure the review. This framework outlines five key stages: (i) identifying the research question, (ii) searching relevant databases, (iii) selecting articles, (iv) charting the findings and extracting key information, and (v) collating, summarising, and reporting the results.

### Research questions and scope

This scoping review aimed to map how women’s empowerment has been addressed in LMICs by examining (i) the conceptual foundations of capacity-building interventions, (ii) the theoretical frameworks used to guide their design and evaluation, and (iii) the indicators applied to measure empowerment and its outcomes. The review specifically sought to answer three questions:

- What are the conceptual foundations of published capacity-building interventions for women’s empowerment or women entrepreneurs in LMICs?
- To what extent, and in what ways, have theoretical frameworks been used to guide the design and evaluation of these interventions?
- What indicators have been used to measure women’s empowerment and its impact in these interventions?

### Eligibility criteria

This scoping review included studies that met the following criteria:

- focused on women entrepreneurs in LMICs;
- examined capacity-building initiatives to enhance entrepreneurial skills through programmes, policies, or interventions;
- used any study design (qualitative, quantitative, or mixed-method), including grey literature, such as reports, policy briefs, and programme evaluations;
- published in English;
- and published between 2010 and 2024.

This timeframe was chosen to capture recent trends and developments and to ensure the results reflect current, rather than past practices to enable appropriate conclusions to be drawn for implications for practice. Entrepreneurship is defined as women’s engagement in income-generating activities that involve business ownership, self-employment, microenterprise development, or participation in structured entrepreneurial support initiatives (e.g. microfinance-linked enterprises, cooperative ventures, digital commerce). Studies where entrepreneurship was either the primary focus or a central mechanism within broader capacity-building interventions were included. Studies addressing broader economic participation were included only where the intervention involved elements consistent with entrepreneurial activity (e.g. business start-up support, income-generating self-employment, enterprise financing, or structured enterprise development training). Pure wage employment or labour-force participation without an enterprise component were excluded.

### Information sources and search strategy

A comprehensive search was carried out across multiple sources. Academic databases included PubMed, Scopus, Web of Science, and JSTOR, while grey literature was searched through websites of organisations engaged in economic development and entrepreneurship in LMICs, such as the World Bank, African Development Bank, and UN Women. This strategy enabled papers from medical, humanities, and social science fields to be accessed and captured programme evaluations which were not published in scientific journals. The full search strategy can be accessed in Supplemental File 1. The search combined keywords and subject headings related to

- women’s empowerment (‘women empowerment theory’)
- capacity-building and training initiatives (‘women entrepreneurs’ OR ‘training programme’ OR ‘skills development’)
- measurement and indicators (‘impact evaluation’ OR ‘effectiveness’ OR ‘program success’)
- theoretical frameworks (‘conceptual model’ OR ‘theory’)
- LMICs (‘Sub-Saharan Africa’ OR ‘Nigeria’ OR ‘South Africa’ OR ‘Tanzania’)

### Study selection process

A total of 11,109 records were identified from all sources. After import into Covidence, a systematic review management tool, 53 duplicates were removed. The use of Covidence streamlined the screening and selection process as it enabled real-time collaboration, and efficient application of inclusion/exclusion criteria to identify relevant studies. Prior to screening, all reviewers participated in three briefing sessions to ensure a shared understanding of the eligibility criteria. A pilot screening of 5% of articles was conducted to assess consistency, with a predefined threshold of >10% disagreement; this threshold was not exceeded.

Screening was conducted in two phases. During both title and abstract and full-text screening, disagreements between reviewers were resolved through discussion, with a third reviewer consulted where consensus could not be reached. Given the scoping review design and emphasis on breadth of inclusion, formal inter-reviewer agreement statistics (e.g. kappa) were not calculated; however, consistency was maintained through independent screening and regular team discussions.

**Figure 1.**
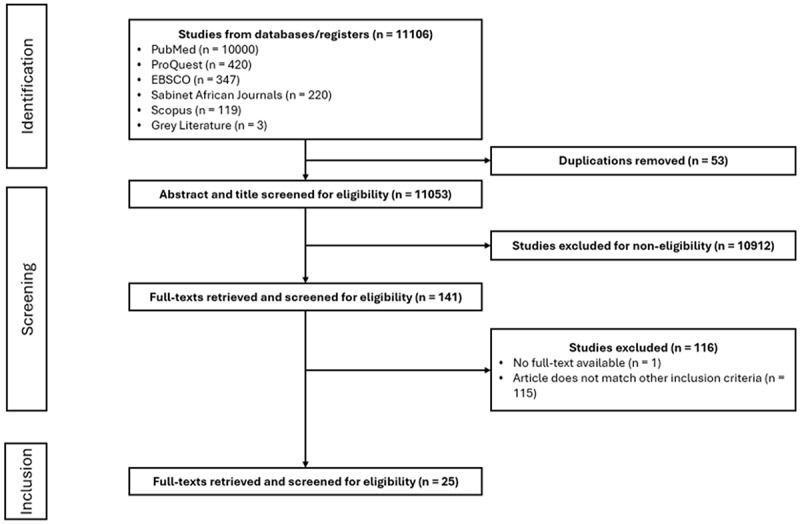
PRISMA-ScR flow diagram of study selection.

This process yielded 141 articles for full-text review. In the second phase, full texts were again screened by two pairs of reviewers (YA and FK; VFC and ACY) with third reviewers resolving conflicts (SB and KS). Ultimately, 25 studies were included in the final review (Figure 1).

Flow diagram illustrating the search and screening process for the scoping review, including the number of records identified, screened, excluded, and retained for analysis (*n* = 25).

### Data extraction

A data extraction form was developed by our team and piloted to ensure consistency. The form was created to include study features relevant to all three papers. Extracted information included: title, author(s), year, country/region, study design, sample size, type of initiative or training, target group, delivery method, duration, content focus, participant profile, target industry/sector, implementation strategy, outcomes, participation barriers, resource constraints, indicators, and recommendations. Data were extracted by SB, cross-verified by YA and AH, and any discrepancies were resolved through consultation with VFC.

### Data synthesis and mapping

Results were mapped thematically and presented in narrative and tabular formats to enable comparison. First, studies were grouped by their focus, study population, and country. Review authors made reasonable assumptions about certain population characteristics if they were not stated explicitly by study authors. For example, socioeconomic groupings were determined by factors, such as loan amount, deprivation of area participant resides in, business revenue, and household income. Next, studies were categorised by how empowerment was described, the constructs applied, and the definitions used. We then examined whether and how theoretical or conceptual frameworks were employed. Finally, empowerment domains and indicators were mapped, with measurement methods and outcomes reported narratively.

## Results

### Study characteristics

Twenty-five studies [36–60] published between 2014 and 2024, representing diverse geographical contexts and research foci were included in this review (Table 1). Most studies focused on economic and employment-related interventions both in the entrepreneurial [36–39,41,43,45–47,49–57] and mixed personal and business support [40,42,44,48,59], with few studies centring health [58] and development [60]. Most studies were conducted in sub-Saharan Africa [37,39,43,46,47,51–54,56,57,59,60] and South Asia [40,43–45,47,48,50,55], with few representing Middle East and North Africa [36,38], South-East Asia [49] and Latin America [41]. Many studies focused exclusively on a rural [45,46,49,52,54,57,58,60] or urban population [39,40,42,47,56,59]; however, the geographical context of participants was often unclear [36–38,41,43,44,50,51]. The age of participants was reported by 68% of studies [36,39–41,43,45–48,52–54,56–59] and large heterogeneity in age groupings was observed. All studies which reported on the age of the participants included women aged 18–65+ [36,39–41,44–48,52–54,56–59], except one which focussed exclusively on women aged over 35 [58]. Socioeconomic background of participants was deemed low in nearly half of the studies [36,37,40,44–46,52,55,57–60], with the remainder including women from various socioeconomic standings [39,41,48,53,56] or not specifying [38,39,42,43,47,49–51,54] this characteristic of their participants.

**Table 1.** Characteristics of included studies.

Characteristic	Type	Subtype	Number of Studies [%]	References
Study focus	Entrepreneurship	Microfinance	5 (20%)	[36,47,49,53,55]
		Social media and radio commerce	2 (8%)	[38,42,43,59]
		General entrepreneurship	6 (24%)	[39,41,45,46,52,54,56,57,60]
		Business support and vocational training	3 (12%)	[45,50,54]
		Social Enterprise and corporate social responsibility	2 (8%)	[37,51]
	Personal and business support	Cash transfers	1 (4%)	[44]
		Social media communication and support groups	2 (8%)	[42,59]
		Financial inclusion	2 (8%)	[40,48]
	Health	Eye health	1 (4%)	[58]
	Sustainable development		1 (4%)	[60]
Settings of population	Rural		8 (32%)	[45,46,49,52,54,57,58,60]
	Urban		6 (24%)	[39,40,42,47,56,59]
	Mixed		3 (12%)	[48,53,55]
	Did not specify/Unknown		8 (32%)	[36–38,41,43,44,50,51]
Countries represented	Nigeria		7 (28%)	[37,39,51,53,54,56,60]
	India		5 (20%)	[40,45,48,50,55]
	Pakistan		2 (8%)	[44,47]
	South Africa		2 (8%)	[52,57]
	Tanzania		2 (8%)	[42,58]
	Ethiopia		1 (4%)	[46]
	Guyana		1 (4%)	[41]
	Indonesia		1 (4%)	[49]
	Jordan		1 (4%)	[38]
	Kenya		1 (4%)	[59]
	Tunisia		1 (4%)	[36]
	Sri Lanka		1 (4%)	[43]

Summary of the 25 studies included in the review, showing their focus, study type, population setting, and country of origin. Studies are grouped by intervention type (entrepreneurship, personal and business support, health, sustainable development) and by population context (rural, urban, mixed, or unspecified). The table also presents the distribution of studies across LMIC regions.

Summary of the 25 studies included in the review, showing their focus, study type, population setting, and country of origin. Studies are grouped by intervention type (entrepreneurship, personal and business support, health, sustainable development) and by population context (rural, urban, mixed, or unspecified). The table also presents the distribution of studies across LMIC regions.

### Conceptualisation of women’s empowerment

Of the included studies, 76% [37–40,42,43,45–54,56,58,59] provided a clear definition of empowerment, while 16% [36,41,44,60] mentioned *empowerment* but did not define it. Two studies [55,57] omitted the term ‘empowerment’ altogether despite describing constructs indicative of empowerment such as decision-making. All studies that used the term *empowerment* distinguished it from *gender equality* [37–40,42,43,45–54,56,58,59]. Empowerment was generally presented as a process or mechanism through which gender equality could be achieved, rather than an interchangeable term.

Across the dataset, we identified a total of 27 distinct definitions of ‘empowerment’ or ‘women’s empowerment’. Additionally, nine definitions of ‘economic empowerment’, three definitions of ‘social empowerment’, and one definition of ‘political empowerment’ were found. Most definitions had been previously published, while four were derived by the authors [43,48,50,54]. Table 2 shows the key constructs considered in the definitions of empowerment quoted by the reviewed studies. Most definitions emphasised decision-making (*n* = 17) and access to resources (*n* = 10), while fewer addressed social justice (*n* = 9), or knowledge and skills (*n* = 5). Some used vague or ill-defined terms, such as ‘*encouraging people to do great things for themselves*’ [54] and ‘*public appearance*’ [53] offering limited conceptual clarity. The definitions of empowerment, their authors and the frequencies they appear in the included studies can be accessed in Supplemental File 2.

**Table 2.** Empowerment constructs in empowerment definitions contained in the dataset.

Description of Empowerment	Empowerment Constructs	Frequency	Definitions Employed in the Studies ( Authors, Year)	Studies Employing a Definition Which Includes the Construct
Agency and decision-making	Goal-setting and strategic decision-making	17	Alsop, 2005 [61] UN, 2011 [62] Kabeer, 2009 [63] Moser, 1989 [64] UN Economic and Social Council, 2002 [65] Kabeer, 1999 [20] Swain, 2007 [66] Abraham and Kalamkar, 2011 [67] Batliwala, 1994 [68] CIDA, 1994 [69] World Bank, 2001 in Krishna, 2003 [70] Jibreel 2018 [54] Kabeer, 2005 [71] Hashemi and Schuler, 1993 [72] Cheston and Kuhn, 2002 [73] Golla et al. 2011 [74] Eyben et al. 2008 [75] Malhotra et al. 2002 [76]	[37,39,40,43,45–47,51,53,54,59]
	Control over life and ability to transform choices into outcomes	13	Alsop, 2005 [61] Keller and Mbewe, 1991 [77] Mosedale, 2005 [25] Rowlands, 1995 [78] UN Economic and Social Council, 2002 [65] Karnavat et al. 2024 [50] Abraham and Kalamkar, 2011 [67] Batliwala, 1994 [68] Chambers, 1993 [79] CIDA, 1994 [69] World Bank, 2001 in Krishna, 2003 [70] Eyben et al. 2008 [75] Alloatti, 2019 [80] Dictionary definition quoted in Rehman et al. 2015 [47]	[38,39,43,46,47,49–51,53,59]
	Autonomy, independence, and self-reliance	4	Keller and Mbewe, 1991 [77] Mosedale, 2005 [25] Rowlands,1995 [78] CIDA, 1994 [69] Jibreel, 2018 [54] Eyben et al. 2008 [75]	[38,39,43,53,54]
	Social and institutional inclusion, having a voice and ability to express opinions	4	Johnson, 1992 [81] Eyben et al. 2008 [75] Sundström et al. 2017 [82] Harvard Kennedy School: Women and Public Policy Program, 2018 [83] Malhotra et al. 2002 [76]	[39,40,43,53]
	Mobility	2	Johnson, 1992 [81] Hashemi and Schuler, 1993 [72]	[40,53]
Material well-being	Access to and control over resources	10	Keller and Mbewe, 1991 [77] Mosedale, 2005 [25] Rowlands 1995 [78] UN, 2011 [62] Moser, 1989 [64] Abraham and Kalamkar, 2011 [67] Chambers, 1993 [79] ODA, 1994 [69] Golla et al. 2011 [74] Sinha et al. 2024 [48] Alloatti, 2019 [80] Brody et al. 2015 [84]	[37–39,43,45,48,49,51–53]
	Economic activity, increase in income, and ability to benefit from economic growth	6	ODA, 1994 [69] Fabiyi and Akande, 2015 [85] Hashemi and Schuler, 1993 [72] Eyben et al. 2008 [75] De Silva and Hansson 2023 [43] Postmus et al. 2013 [86]	[39,40,48,53,54]
	Ability to meet needs and improve living standards	4	Chambers, 1993 [79] Al-Dajani and Marlow, 2013 [87] Parwez and Patel, 2022 [88] Shuja et al. 2020 [89] Swain and Wallentin, 2009 [90]	[48,49,53]
Influence, rights, and power	Ability to influence social change and assert rights	10	Keller and Mbewe, 1991 [77] Mosedale, 2005 [25] Rowlands, 1995 [78] UN, 2011 [62] Kabeer, 2009 [63] Moser, 1989 [64] Karnavat et al. 2024 [50] Jatana and Crowther, 2007 [91] Batliwala, 1994 [68] Batliwala 1994, in Parpart et al. 2002 [92] Jibreel, 2018 [54] Sundström et al. 2017 [82] Harvard Kennedy School: Women and Public Policy Program, 2018 [83]	[37,38,40,42,43,45,50,51,53,54,59]
	Power, authority, and a strong position in the family and society	6	Abraham and Kalamkar, 2011 [67] Batliwala 1994, in Parpart et al. 2002 [92] Acosta-Belen and Bose, 1990 [93] Stark et al. 2018 [94] Swain and Wallentin, 2009 [90] Dictionary definition quoted in Rehman et al. 2015 [47] Malhotra et al. 2002 [76]	[40,43,47,51,53]
	Involvement in political affairs	1	Sundström et al. 2017 [82] Harvard Kennedy School: Women and Public Policy Program, 2018 [83]	[40]
Social justice	Gender equality in the distribution of wealth, power, and participation	7	Keller and Mbewe, 1991 [77] Mosedale, 2005 [25] Rowlands 1995 [78] Kabeer, 2009 [63] Karnavat et al. 2024 [50] Jatana and Crowther, 2007 [91] Sinha et al. 2024 [48] Alloatti, 2019 [80] Al-Dajani and Marlow, 2013 [87] Parwez and Patel, 2022 [88] Shuja et al. 2020 [89]	[38,42,43,48–51]
	Recognition of the value of women’s contributions	1	Fabiyi and Akande, 2015 [85]	[54]
	Respecting women’s dignity	1	Fabiyi and Akande, 2015 [85]	[54]
Personal capacities	Capacity, knowledge, and skills	5	Sawe, 2021 [95] Solomon, 1976 [96] CIDA, 1994 [69] Alloatti, 2019 [80] Postmus et al. 2013 [86]	[48,49,53,58]
	Motivation, sense of self-assurance, and self-worth	4	UN, 2011 [62] Solomon, 1976 [96] CIDA, 1994 [69] Eyben et al. 2008 [75]	[37,39,53,58]

Compilation of empowerment constructs and keywords appearing in definitions quoted by the reviewed studies. Constructs are grouped into thematic domains such as Agency and Decision-Making, Material Well-Being, Influence, Rights and Power, Social Justice, and Personal Capacities. Frequency describes the number of times the empowerment constructs are mentioned in the definitions employed by the studies. Some definitions include multiple key words (eg. influence, social change) associated with the construct (eg. ability to influence social change and assert rights) which were counted separately. In some studies, several definitions mentioned the same construct resulting in several counts of the same key word.

Compilation of empowerment constructs and keywords appearing in definitions quoted by the reviewed studies. Constructs are grouped into thematic domains, such as agency and decision-making, material well-being, influence, rights and power, social justice, and personal capacities. Frequency describes the number of times the empowerment constructs are mentioned in the definitions employed by the studies. Some definitions include multiple keywords (e.g. influence, social change) associated with the construct (e.g. ability to influence social change and assert rights) which were counted separately. In some studies, several definitions mentioned the same construct resulting in several counts of the same keyword.

### Use of theoretical or conceptual frameworks

Fifteen (60%) of the reviewed studies literature did not explicitly mention the application of any empowerment framework during the design, implementation, or interpretation stages of their interventions [37,39–41,44,45,48–52,54,55,57,60]. Among the 10 studies that used a framework, six applied published frameworks without modification [42,46,53,56,58,59], three developed their own context-specific models [36,38,43] and one adapted an existing framework [47]. Notably, each study used a different framework. Table 3 provides an overview of the frameworks identified and outlines how they were applied within the respective studies. It was not possible to determine how one study used the framework it described [42].

**Table 3.** Frameworks and outlines on their applications in the included respective studies.

Framework	Description of Framework	How Framework Is Used		Employed in Study
		Study Design	Evaluation of Results	
Longwe [97]	Empowerment is described as five levels in ascending order from welfare to access, conscientisation, participation, and control.	Yes	Yes	[59]
Mandal’s five elements of women empowerment [98]	Empowerment is split into five categories: social, psychological, educational, economic and political.	Yes	No	[58]
Empowerment theories by Solomon, 1976 [96] and Batliwala, 1994 [68]	Personal, interpersonal, and environmental resources are viewed as necessary to increase and improve the skills, knowledge, and motivation of people to achieve valid social roles and power.	Yes	No	[56]
Kabeer [20]	Empowerment is viewed through three dimensions: access to resources, agency, and achievements.	No	Yes	[46]
Theory of Digital Technologies and Women empowerment by Lechman and Paradowski, 2021 [99]	Digital communication technologies are seen as key drivers for women’s empowerment and government policy interventions to support skills development are important to enable empowerment.	Not reported	Not reported	[42]
Framework for microfinance and women empowerment adopted from Malhotra, Schuler and Boender, 2002 [22]	Empowerment is described in six dimensions: economic, socio-cultural, familial/interpersonal, legal, political, and psychological with indicators used at different levels of social aggregation.	Yes	Yes	[53]
Framework adapted from *Reaching and empowering women IFAD*, by Mayoux and Hartl, 2009 [100]	Three paradigms [feminist empowerment paradigm, poverty alleviation and financial sustainability paradigm] are used to describe empowerment.	Yes	Yes	[47]
De Silva and Hansson [43]	Describes a resource and capabilities view of empowerment.	Yes	Yes	[43]
Al-Omoush and Al-Qirem [38]	Describes women hedonic, women utilitarian, self-efficacy, and social support factors which lead to social commerce as a tool for women innovation.	Yes	Yes	[38]
Fitouri and Zouaoui [36]	Describes saving practice, skill development, business support, access to finance and microfinance services as tools for women’s entrepreneurship development.	Yes	No	[36]

List of the theoretical or conceptual frameworks explicitly employed in the reviewed studies, outlining the main characteristics of each framework and whether it was applied in intervention design and/or evaluation. Frameworks include Kabeer’s resources–agency–achievements model, Longwe’s five-level empowerment framework, and others adapted or developed locally.

List of the theoretical or conceptual frameworks explicitly employed in the reviewed studies, outlining the main characteristics of each framework and whether it was applied in intervention design and/or evaluation. Frameworks include Kabeer’s resources–agency–achievements model, Longwe’s five-level empowerment framework, and others adapted or developed locally.

### Empowerment domains and indicators

**Figure 2.**
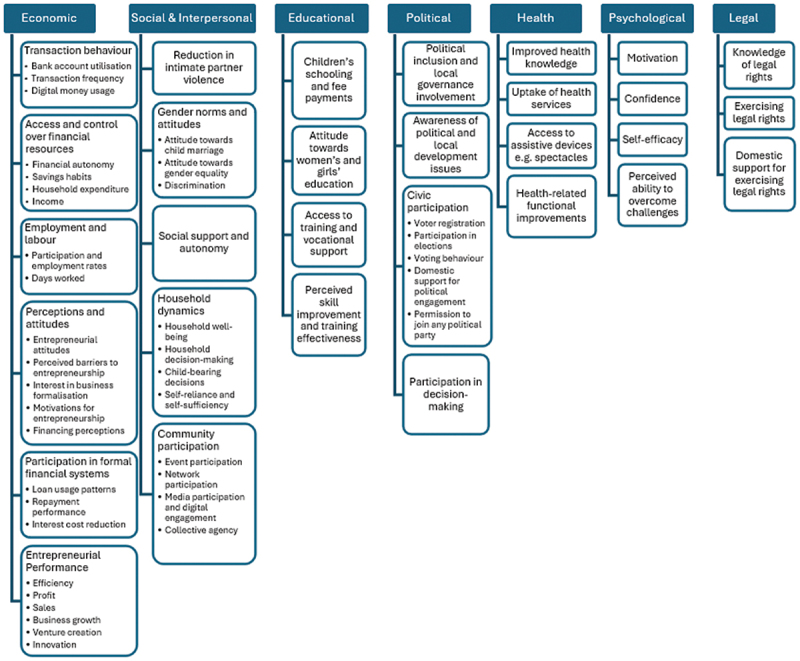
Domains of empowerment measured across included studies.

All reviewed studies assessed economic empowerment [36–60], making it the most consistently evaluated domain. Social empowerment was explored by 11 (44%) studies [37–40,46–48,51,53,58,59], while educational [39,45,53,56,58] and political empowerment [40,46,47,53,58] were considered by 5 (20%) studies each. A smaller number of studies addressed psychological (16%) [38,53,58,59], health (8%) [51,58], and career-related (4%) [51] empowerment. Only two studies considered legal empowerment [46,53], and one study considered familial or interpersonal empowerment [50] separately from social empowerment. Thematic mapping of indicators revealed substantial heterogeneity in how empowerment was operationalised across studies. Indicators used were relevant to the domain of empowerment examined in all studies. However, three studies [47,49,52] used increase in income and profit as a proxy measure to examine financial decision-making ability, assuming that these concepts are interchangeable (Figure 2).

Graphical representation of the frequency with which different empowerment domains: economic, social, political, educational, psychological, health, legal, and interpersonal were assessed among the included studies. Economic empowerment was the most commonly measured domain.

### Study design, measurement methods, and outcomes

All studies were cross-sectional [36–43,45–60], except one which collected responses from participants and a control group at baseline, 2, 5, and 8 years into the intervention [44]. Six (24%) studies used mixed-method design [43,46,49,52,55,60], nine (36%) were qualitative [37,39,41,42,45,47,51,58,59], and the remainder were quantitative [36,38,40,44,48,50,53,54,56,57]. Eleven studies [36,38,40,43,48,49,52–54,56,57] used validated tools, and 14 used self-developed tools without explicitly mentioning their validation before employment [37,39,41,42,44–47,50,51,55,58–60]. Two studies [46,51] used group-level measurement with the remainder examining individuals [36–45,47–50,52–60].

Seven studies (28%) reported long-term outcomes only [41–43,45,46,49,60] and one study reported short- and long-term empowerment outcomes [44]. Most studies (68%) did not specify whether they measured long- or short-term outcomes [36–40,47,48,50–59]. Outcomes were classed as short-term or long-term based on study author descriptions. If this was not available, review authors categorised the outcomes as short-term, if follow-up or impact were at 2 years or less. Fourteen studies did not report impact of intervention based on any demographic variables [36–41,43,45,47–49,54–60]. One study reported results disaggregated by age, schooling, occupation, marital status, presence of polygamy and a joint family (defined as living with husband’s relatives) [53]. One study explored the relationship between entrepreneurship and age, education, and years of experience [50]. Others chose to present outcomes based on socioeconomic status [44], by number of days worked [52], by bank used [51], and by region [46]. Most studies used exclusively self-reported indicators which described participants’ perceived empowerment (e.g. perceived skill improvement, self-efficacy, motivation, income stability) without a quantitative or binary measure [36]. Three studies used objective indicators (e.g. weekly income, loan repayment record) only [48,51,55], and four used both self-reported and objective indicators [49,52–54]

## Discussion

This scoping review assessed whether and how capacity-building interventions in LMICs define and measure women’s empowerment, and whether and how they are guided by theoretical or conceptual frameworks. We found wide variation in definitions, limited and inconsistent use of frameworks, and a strong focus on economic empowerment indicators.

### Conceptualisation of women’s empowerment

As Yount et al. [101] pointed out, Sustainability Development Goal 5 (SDG5) monitoring requires better measurement whereby women’s empowerment must be clearly defined, adequately measured with representative samples, and comparable across countries, years, and social groups. Our review highlights a persistent dilemma in measuring women’s empowerment. Most included studies provided a definition of empowerment, even though they varied widely from decision-making [37,39,40,43,45–47,51,53,54,59] and access to resources [37–39,43,48,49,51–53], to social justice [38,42,43,48–51], autonomy [38,39,43,53,54], or personal confidence [37,39,53,58]. Several linked empowerments to political participation [40] or legal rights [46,53]. On the one hand, standardisation is necessary to enable comparison across studies, contexts, and time, and to provide policymakers with evidence that can inform global monitoring frameworks such as SDG5. On the other hand, empowerment is inherently multidimensional and context-specific. Over-standardisation risks flattening these nuances and overlooking what empowerment means in cultural or social settings. A balanced approach is therefore needed: adopting a set of core indicators that allow for comparability, while simultaneously incorporating context-specific measures that reflect local understandings and priorities. For example, core indicators might include women’s role in household decision-making, access to income or property, participation in paid work, and freedom of movement, while context-specific measures could capture dimensions such as political participation, digital skills for entrepreneurship, agricultural decision-making, or psychological empowerment (e.g. confidence and critical awareness). Such an approach would strengthen both the conceptual clarity and the practical relevance of empowerment research.

It was also noted that economic aspects of empowerment were often emphasised [36–60]. Increase in income is often seen instrumentally as a mediator or catalyst to achieve economic growth, rather than as an indicator of increasing women’s rights and agency in multiple domains. While studies [102,103] have shown the positive impact of economic improvement in empowerment, we risk reducing the influence of structural barriers, such as restrictive gender and cultural norms and systemic exclusion by equating empowerment with income improvement alone. However, Bayissa’s study [104] shows that the psychological aspect is the most pivotal in linking the six dimensions of empowerment, while the economic dimension shows minimal connection, suggesting that a comprehensive set of indicators is required to advance empowerment across all areas. Hence, we agreed with Duflo [23] that while women’s empowerment and economic development are mutually reinforcing, the link is not strong enough to sustain itself; lasting gender equality requires continuous, deliberate policy commitment rather than reliance on a self-sustaining virtuous cycle.

### Use of theoretical or conceptual frameworks

Few studies explicitly described the frameworks to guide their design or evaluation. This could be interpreted in two ways. One, these studies were mainly qualitative or explorative in nature and deliberately avoided predefined frameworks to let concepts emerge. Two, findings from these studies reported, mainly patterns, rather than mechanisms. Where frameworks were used, they were highly diverse. Again, this inconsistency shows that empowerment is context-specific. It could be operationalised differently in different settings. However, the absence of shared reference points makes it difficult to build cumulative knowledge.

### Empowerment domains and indicators, measurement methods, and outcomes

The measurement of empowerment outcomes was also inconsistent. The findings show that all included studies assessed economic empowerment [36–60] and a few examined social [37–40,46–48,51,53,58,59], political [40,46,47,53,58], legal [46,53], or health-related domains [51,58]. We hypothesise that it is because economic empowerment is a measurable outcome that is seen to reflect improvement in well-being and shaped by global development agenda that prioritise productivity and income growth. However, we recommend that a shift to measure autonomy, social, cultural, and political components is required to provide a more holistic view of women’s empowerment.

We found that many studies used self-reported indicators [36–47,49,50,52–54,56–60] (e.g. confidence, satisfaction, or perceived decision-making power). These indicators add insights into personal experiences and human agency on top of economic empowerment; however, they also make it more difficult to compare the effect size between interventions. Few studies used combined subjective and objective measures which limited the breadth of their measurement of empowerment [43]. Many studies [37,39,41,42,44–47,50,51,55,58–60] used nonvalidated tools and proxy indicators, such as income and profit as stand-ins for empowerment, assuming that financial gain automatically leads to increased decision-making power, weakening construct validity.

Another limitation is that most studies collected data at a single point in time [36–43,45–60]. Empowerment is widely recognised as a process that evolves, yet longitudinal studies that included multiple and longer follow-up were rare. Without tracking changes over time, it is difficult to know whether reported improvements are sustained or whether they reflect short-term changes only. Furthermore, few studies disaggregated their results by factors, such as age, marital status, or education. When studies treat ‘women’ as a single, homogenous category, they fail to capture differences across subgroups (e.g. by age, marital status, education, class, caste, ethnicity, disability, or rural/urban location) [20]. This limits understanding of how empowerment is experienced differently. For example, younger unmarried women may have very different constraints than older married women. It also risks masking inequalities within the broad group ‘women’, with individual circumstances lost in reported averages.

### Implications

This review underscores the need for clarity and rigour in women’s empowerment research and practice. For researchers, it highlights the importance of explicitly defining empowerment, stating the frameworks applied both in the study design and evaluation of results and employing validated tools where possible. Indicators used should show effect size, be comparable, yet context-specific, and represent multiple dimensions of empowerment. Mixed-methods and longitudinal approaches may offer richer insights into empowerment as an evolving process. However, we acknowledge that longitudinal design and the use of published frameworks, although beneficial, may not be feasible or suitable in all contexts based on the intervention type and population studied and recognise challenges that may arise, including resource limitations, capacity gaps, data infrastructure constraints and political and institutional barriers. For practitioners and policymakers, the findings caution against equating income or skills training with empowerment, stressing the need to specify which dimensions are being targeted and to measure them accordingly to ensure high construct validity.

### Limitations

The review’s limitations must be acknowledged. We only included English-language publications, which may have excluded relevant evidence, and the heterogeneity of studies, and limited synthesis into a unified framework. In addition, as a scoping review, the focus was on mapping and describing the literature rather than assessing the effectiveness of interventions. However, these will be reported separately.

## Conclusions

To our knowledge, this is the first scoping review to systematically examine not only what capacity-building interventions for women entrepreneurs in LMICs do, but how empowerment is conceptually framed, theoretically grounded, and empirically measured within them. By revealing substantial heterogeneity, limited theoretical anchoring, and over-reliance on economic proxies, this review highlights a critical gap between empowerment theory and implementation practice. Moving forward, research should prioritise theory-driven programme design, multidimensional and context-sensitive measurement frameworks, longitudinal evaluation strategies, and intersectional disaggregation to better capture empowerment as a dynamic and relational process. Bridging conceptual rigor with implementation feasibility will be essential to ensure that capacity-building interventions contribute meaningfully to gender equity and the Sustainable Development Goals.

## Supplementary Material

Supplemental File 2 Definitions employed by the included studies.docx

Supplemental File 1 Search strategy for scoping review.docx

## Data Availability

All data are included in the manuscript.
