# Supplementary material for: A scoping review of theoretical and measurement approaches to women’s empowerment in low-and middle-income countries’ capacity-building interventions
Source: Glob Health Action. 2026 May 26;19(1):2676412. doi: 10.1080/16549716.2026.2676412 (PMC13215425; doi:10.1080/16549716.2026.2676412)
Supplement: Supplemental File 2 Definitions employed by the included studies.docx [file ZGHA_A_2676412_SM3537.docx]

## Supplemental File 2: Definitions of empowerment used in included studies

| **Definition** | **Author and Date** | **Frequency** |
| --- | --- | --- |
| ‘Empowerment’ Definitions | | |
| The process of enhancing an individual’s or group’s capacity to make purposive choices and to transform these choices into desired actions and outcomes. | Alsop, 2005 | 2 |
| Individual capability of women to increase their self-reliance, organize themselves, support their rights to transform personal interests and choices into desired outcomes, and control resources to eliminate their subordination. | Keller & Mbewe, 1991; Mosedale 2005; Rowlands 1995 | 2 |
| Empowerment comprises five major components: women’s sense of self-worth; right to determine choices; right to access opportunities and resources; power to control own lives; and ability to influence the direction of social change to create a more just social and economic order nationally and internationally. | UN, 2011 | 1 |
| The processes of change through which women expand their ability to make strategic choices about their lives and to participate on equal terms with men in bringing about desired changes in the society in which they live. | Kabeer, 2009 | 1 |
| The right to determine choices in life and to influence the direction of change through the ability to gain control over crucial material and nonmaterial resources. | Moser, 1989 | 1 |
| The process by which women take control over their lives, acquiring the ability to make strategic choices. | UN Economic and Social Council, 2002 | 1 |
| To give somebody the power or authority to do something or to give somebody more control over their life or the situation they are in. | Dictionary definition quoted by Rehman et al, 2015 | 1 |
| A process by which those who have been denied the ability to make strategic life choices acquire such ability. | Kabeer 1999; Swain, 2007 | 1 |
| Efforts to increase capacity, especially for vulnerable groups. | Sawe, 2021 | 1 |
| Women empowerment is a multifaceted concept encompassing social, economic, and political dimensions aimed at creating an environment where women can exercise autonomy, assert their rights, and participate equally in society. | Karnavat et al, 2024 | 1 |
| Process of change by which individuals or groups gain power and ability to take control over their lives. It involves access to resources, resulting in increased  participation in decision-making and bargaining power and increased control over benefits, resources and own life. | Abraham and Kalamkar, 2011 | 1 |
| The process by which unequal power relations between men and women are transformed, allowing women to gain equality with men and, thereby, challenge social norms and restrictions to foster development. | Jatana and Crowther, 2007 | 1 |
| To increase and improve the skills, knowledge and motivation of people to achieve valid social roles. | Solomon, 1976 | 1 |
| The ability of women to influence external actions relating to their welfare and well-being. In this case, empowerment means that women gain control, and the capacity to make choices individually or collectively, and then transform these choices into desired outcomes. | Batliwala, 1994 | 1 |
| Having a voice, freedom of movement and public appearance. | Johnson, 1992 | 1 |
| A situation whereby people, particularly the poor, are supported in terms of taking charge of their lives and securing a better living standard, which will include owning and controlling productive resources as basic component. | Chambers, 1993 | 1 |
| People taking charge of their lives, defining their own goals, solving their own problems, acquiring productive skills, establishing self-reliance and building their self-assurance. | CIDA, 1994 | 1 |
| A process which enables people to act collectively to enhance their access to public services and increase their gains from the process of economic growth. | ODA, 1994 | 1 |
| A process whereby people challenge the prevailing power structures to increase their control of power from its source. | Batliwala, 1994 in Parpart et al. 2002 | 1 |
| A process which helps to improve women’s capabilities in making choices that will translate to their preferred actions and results. | World Bank, 2001 in Krishna, 2003 | 1 |
| The capacity of women to participate in, contribute to and benefit from growth processes in ways which recognize the value of their contributions, respect their dignity and make it possible to negotiate a fairer distribution of the benefits of growth. | Fabiyi and Akande, 2015 | 1 |
| The process whereby people gain the ability and authority to make informed decisions and implement change in their own lives and the lives of other people. It is a means of encouraging people to do great things for themselves and also to make great impact in their society. | Jibreel, 2018 | 1 |
| Women’ ability to make life choices. | Kabeer, 2005 | 1 |
| Mobility, economic activity, and decision-making power. | Hashemi and Schuler, 1993 | 1 |
| Restructuring power relations. | Acosta-Belen and Bose,1990 | 1 |
| Advancing participation in decision-making in all spheres of life | Cheston and Kuhn, 2002 | 1 |
| Empowerment is an improvement in a person’s conditions from having less power to more power, providing the opportunity for social inclusion and the ability to make life choices | Malhotra et al, 2002 | 1 |
| The capacity to make and work on decisions that involve the control over and allocation of financial resources and profits | Golla et al, 2011 | 1 |
| The development of a reasoning capability that looks beyond immediate daily survival but rather a thought and action process that allows individuals exercise a higher level of influence and control over both their assets and life choices, decisions made around making investments and seizing opportunities in order to increase their income. | Eyben et al, 2008 | 1 |
| The ability to contribute to growth processes in a way that recognises the worth of their work and ensures a just distribution of their wealth to increase access to economic resources. | Sinha et al, 2024 | 1 |
| Women’s efforts to increase their ability to be equally empowered as men, especially in terms of access to productive resources, to be able to participate in the utilization of these productive resources, have the same control over productive resources as men, have an equal distribution from the use of existing productive resources, including national economic development output, and have equal financial literacy | Alloatti, 2019 | 1 |
| Increase the community’s ability to meet their basic needs and gain equal access. | Al-Dajani and Marlow 2013; Parwez and Patel 2022; Shuja et al, 2020 | 1 |
| An individual’s ability to access resources, own resources and control such resources. | Brody et al. 2015 | 1 |
| Performing business which leads to overall empowerment of a woman. | De Silva and Hansson, 2023 | 1 |
| Enhancement of women’s capacity for strategic choice and agency in the sphere of the economy and to the possibilities this opens up for change in other spheres of their lives. | Kabeer, 2009 | 1 |
| Economic empowerment includes financial literacy, which provides the knowledge and skills to manage financial prosperity, economic freedom, and responsibility for finances. | Postmus et al, 2013 | 1 |
| Social empowerment entails the act of creating a sense of independence, increased self-esteem, collective action or singularly ensuring that social interactions and institutions do not leave out those who are perceived as  impoverished people and keep them in penury. | Eyben et al, 2008 | 1 |
| Giving someone the ability to better their standard of living. | Swain and Wallentin, 2009 | 1 |
| Key enablers that strengthen women’s social relations and position in the family and society. | Stark et al, 2018; Swain and Wallentin, 2009 | 1 |
| The ability to be involved in decision-making in the social sphere, express opinion voluntarily and involvement in political affairs. | Sundström et al, 2017; Harvard Kennedy School: Women & Public Policy Program, 2018 | 1 |
