# Supplementary material for: A scoping review of theoretical and measurement approaches to women’s empowerment in low-and middle-income countries’ capacity-building interventions
Source: Glob Health Action. 2026 May 26;19(1):2676412. doi: 10.1080/16549716.2026.2676412 (PMC13215425; doi:10.1080/16549716.2026.2676412)
Supplement: Supplemental File 1 Search strategy for scoping review.docx [file ZGHA_A_2676412_SM3536.docx]

Supplemental File 1: Search strategy for the scoping review

| (“capacity building” OR “training program” OR “entrepreneurial education” OR “skills development” OR “entrepreneurship training” OR “business training” OR “empowerment program”)  AND  (“women entrepreneurs” OR “female entrepreneurs” OR “women-owned business” OR “female-led enterprise” OR “women entrepreneurship” OR “female business owners”)  AND  (“Sub-Saharan Africa” OR “SSA” OR "Africa South of Sahara" OR “Nigeria” OR “Kenya” OR “South Africa” OR “Uganda” OR “Ghana” OR “Ethiopia” OR “Tanzania” OR “Zambia” OR “Rwanda” OR “Mozambique”)  AND  (“outcome measurement” OR “impact evaluation” OR “effectiveness” OR “program success” OR “outcomes” OR “indicators”)  AND  (“theoretical framework” OR “theory” OR “conceptual model” OR “entrepreneurship theory” OR “women empowerment theory”) |
| --- |
